# Stratified primary care versus non-stratified care for musculoskeletal pain: findings from the STarT MSK feasibility and pilot cluster randomized controlled trial

**DOI:** 10.1186/s12875-019-1074-9

**Published:** 2020-02-11

**Authors:** J. C. Hill, S. Garvin, Y. Chen, V. Cooper, S. Wathall, B. Saunders, M. Lewis, J. Protheroe, A. Chudyk, K. M. Dunn, E. Hay, D. van der Windt, C. Mallen, N. E. Foster

**Affiliations:** 1 Primary Care Centre Versus Arthritis, School of Primary, Community and Social Care, Keele University, Keele, Staffordshire ST5 5BG UK; 2 Keele Clinical Trials Unit, School for Primary, Community and Social Care, Faculty of Medicine and Health Sciences, Keele University, Newcastle, UK

**Keywords:** Musculoskeletal pain, Stratified care, Prognosis, Primary care, General practice

## Abstract

**Background:**

Musculoskeletal (MSK) pain from the five most common presentations to primary care (back, neck, shoulder, knee or multi-site pain), where the majority of patients are managed, is a costly global health challenge. At present, first-line decision-making is based on clinical reasoning and stratified models of care have only been tested in patients with low back pain. We therefore, examined the feasibility of; a) a future definitive cluster randomised controlled trial (RCT), and b) General Practitioners (GPs) providing stratified care at the point-of-consultation for these five most common MSK pain presentations.

**Methods:**

The design was a pragmatic pilot, two parallel-arm (stratified versus non-stratified care), cluster RCT and the setting was 8 UK GP practices (4 intervention, 4 control) with randomisation (stratified by practice size) and blinding of trial statistician and outcome data-collectors. Participants were adult consulters with MSK pain without indicators of serious pathologies, urgent medical needs, or vulnerabilities. Potential participant records were tagged and individuals sent postal invitations using a GP point-of-consultation electronic medical record (EMR) template. The intervention was supported by the EMR template housing the Keele STarT MSK Tool (to stratify into low, medium and high-risk prognostic subgroups of persistent pain and disability) and recommended matched treatment options. Feasibility outcomes included exploration of recruitment and follow-up rates, selection bias, and GP intervention fidelity. To capture recommended outcomes including pain and function, participants completed an initial questionnaire, brief monthly questionnaire (postal or SMS), and 6-month follow-up questionnaire. An anonymised EMR audit described GP decision-making.

**Results:**

GPs screened 3063 patients (intervention = 1591, control = 1472), completed the EMR template with 1237 eligible patients (interventio*n* = 513, control = 724) and 524 participants (42%) consented to data collection (interventio*n* = 231, control = 293). Recruitment took 28 weeks (target 12 weeks) with > 90% follow-up retention (target > 75%). We detected no selection bias of concern and no harms identified. GP stratification tool fidelity failed to achieve a-priori success criteria, whilst fidelity to the matched treatments achieved “complete success”.

**Conclusions:**

A future definitive cluster RCT of stratified care for MSK pain is feasible and is underway, following key amendments including a clinician-completed version of the stratification tool and refinements to recommended matched treatments.

**Trial registration:**

Name of the registry: ISRCTN. Trial registration number: 15366334.

Date of registration: 06/04/2016.

## Background

Musculoskeletal (MSK) pain from common conditions such as back pain and osteoarthritis are costly global health challenges, particularly for primary care where the majority of patients are managed. For example, in the UK, common MSK problems such as back, shoulder, knee and multi-site pain account for 14% of General Practitioner (GP) consultations [1] and estimates from the most recent global burden of disease studies suggest they are the leading cause of disability adjusted life years (DALYs) [2, 3]. Given the ageing population and the increasingly complex and multi-morbid clinical presentations of patients, clinical decision-making is becoming more challenging [4–6]. In addition, consultation rates for MSK pain are increasing, for example in the UK, GP consultations for MSK pain have increased by 19% (from 310 to 370 million per year) over a five-year period [7, 8].

Randomised controlled trials (RCTs) show that non-pharmacological interventions such as physiotherapist-led supervised exercise and cognitive behavioural approaches are more effective than minimal usual care [9–12], yet most guidelines [13–15] lack clarity about which patients should be offered these additional interventions [16–18]. At present, primary care decision-making for MSK pain is mostly based on ruling out serious pathology and using clinical reasoning without formal stratification tools to decide on treatment. Assessing the severity, impact and prognosis of individual patients can be difficult in short primary care consultations and patient access to other treatments is often variable [19–22]. Offering everyone consulting in primary care with MSK pain further treatments is both unnecessary and impractical [16, 17]. Therefore, finding ways to better identify which patients to de-medicalise by limiting care primarily to reassurance and self-management whilst conversely identifying which patients should be offered more intensive and expensive healthcare treatments, is an international priority [14, 17, 23].

We have previously demonstrated the clinical- and cost-effectiveness of a stratified primary care approach to support clinical decision-making for patients with low back pain in the UK [24–26]. This approach combines prognostic stratification (using the STarT Back tool that classifies individuals into either a low, medium or high risk subgroup for persistent low back pain-related disability) with recommended matched treatments for each subgroup [27–29]. This approach to stratified care for low back pain has since been recommended in several international clinical guidelines [30–32]. Whilst low back pain is the most common MSK pain presentation in primary care, it accounts for only 26% of the MSK caseload [1], and it is unknown whether a similar prognostic approach to stratified care would benefit the large volume of patients with MSK pain in other body sites/locations (e.g. knee or shoulder pain).

Given the results of several systematic reviews showing consistent prognostic factors across MSK pain conditions [33–37], we developed and validated a single prognostic stratification tool, the Keele STarT MSK tool, for use among patients with the five most common MSK pain presentations in primary care (back, neck, shoulder, knee, and multi-site pain) [1]. The Keele STarT MSK Tool has shown good predictive and discriminative ability in development and validation samples [38], identifying patients at low, medium or high risk of persistent MSK pain over 6-months. Using systematic review and consensus methods, we also agreed evidence-based recommended matched treatment options for each of the risk subgroups [39, 40].

The STarT MSK stratified primary care intervention has two components: use of the tool to identify risk subgroups, followed by matched treatment options. A definitive trial is needed to test whether this approach is better for patients’ outcomes and the healthcare system, compared to usual non-stratified care. Prior to conducting the main randomised controlled trial (RCT), we examined the feasibility of a) a future definitive cluster RCT, and b) GPs using stratified care at the point-of-consultation. Specific objectives were to:

1. Estimate participant recruitment and follow-up rates in a pilot cluster RCT
2. Examine evidence of selection bias between trial arms and participants and non-participants
3. Assess GP fidelity to the stratified care intervention (use of the stratification tool and matched treatments) at the point-of-consultation.
4. Conduct secondary descriptive analyses of GP decision-making and patient self-reported outcomes.

## Methods

### Trial design

The study design was a pragmatic, feasibility and pilot, two-parallel arm (1:1 ratio), cluster RCT in 8 general practices, with a nested qualitative study reported separately [41]. A cluster RCT was chosen over an individual patient randomisation design as stratified MSK care involves GPs using a slightly different consultation approach following specific training, as well as the use of a bespoke electronic medical record (EMR) template, which was only possible to implement at a practice level without causing a high probability of intervention contamination across arms [42]. The units of randomisation were the general practices and units of observation were adults consulting with MSK pain. The International Standard Randomised Controlled Trials Number is ISRCTN15366334.

### Participant eligibility criteria and identification

Patients were eligible if, during their visit to a participating GP practice, the trial’s purpose-built participant identification screen, embedded within the EMR, was completed at the point-of-consultation, including GP confirmation of patient eligibility. Inclusion criteria were: aged over 18 years, registered at that general practice, consulting for MSK pain in the back, neck, shoulder, knee or multi-site pain. The trial identification template activated automatically for all new or returning episode cases when GPs (intervention and control) entered one of over 200 pre-identified MSK Read-codes (i.e. symptom/ diagnostic codes) into the patient’s electronic medical record (EMR). Exclusions were: clinical indicators of (suspected) serious ‘red flag’ pathology requiring urgent medical intervention or a known systemic inflammatory condition, those unable to communicate in English (both in reading and speaking), vulnerable patients including those on the ‘severe and enduring mental health register’, a diagnosis of dementia or terminal illness, and recent trauma or bereavement. To reduce patient/clinician burden, the participant identification screen only activated once per patient (providing it was completed or an exclusion was entered). A further eligibility criterion, administrated by the research centre, specified that initial questionnaire responses were completed within 4 weeks of invitation mailing date (using self-reported date-of-completion on the questionnaire).

### Recruitment

#### General practices

The UK West Midlands National Institute for Health Research (NIHR) Clinical Research Network (CRN) facilitated recruitment of eight general practices who used the EMIS Web EMR system and collectively served a target population of > 40,000 adults. GP practice eligibility criteria included willingness to be randomised to either stratified care or usual care, to engage in intervention training (if allocated to stratified care) and to facilitate an anonymised EMR audit after 6-months in the trial. Practices were also required to remove any existing MSK stratification tools (e.g. STarT Back) if they were randomised as a control practice. Consent to these criteria was sought through a written agreement with a representative from each participating practice, prior to randomisation. We aimed for practices that varied in size, location (urban, semi-urban and rural) and population socio-demographics.

#### Patients

Patient identification, invitation and recruitment were facilitated by CRN staff, or practice staff (if preferred), through a weekly download into a secure mailing database of eligible patients identified from the trial’s IT identification template. Eligible patients were sent a study invitation letter and information leaflet, an initial questionnaire and a consent form with a stamped addressed envelope to return. A study administrator (blind to GP practice allocation) was available for telephone support if required. Signed consent to provide questionnaire outcome data was obtained from all participants and NHS ethical approval gained (Reference: 16/EM/0257). Participant recruitment lasted 8 months (October 2016 to May 2017).

### Randomisation and blinding

Randomisation used stratified block randomisation based on GP practice list size to allocate the 8 practices in a ratio of 1:1 (4 intervention, 4 control). Keele Clinical Trials Unit (CTU) computer-generated the random sequence and ensured concealment by providing each practice with an anonymised code. Allocation (at cluster and individual level) was shared with the study team (except for the trial statistician and outcome data collectors who were blinded until the analysis was finalised). Blinding for participating GPs was obviously not possible, however, patients were unaware of the RCT and the differences between consultations in intervention and control practices, and instead were informed about, and consented to, providing questionnaire data for a study investigating the Treatment of Aches and Pains (TAPs). These processes follow recommendations for cluster RCTs [42].

### Interventions

#### Usual care

Patients consulting at the four usual care general practices received clinical care as usual for MSK pain. Usual primary care is known to be variable [43–45]; for example, some patients may receive advice, prescriptions for medications and nothing more, some may be asked to return to the GP for follow-up assessment or treatment, whereas others may be referred to other services, including for tests and investigations, or treatment services such as physiotherapy, orthopaedics or pain clinics. As part of the trial’s participant identification screen, GPs in control (and intervention) practices recorded patient’s average MSK pain intensity (see outcomes section) and primary MSK pain site at the point-of-consultation on the study EMR identification template.

#### Stratified care intervention

**Fig. 1:**
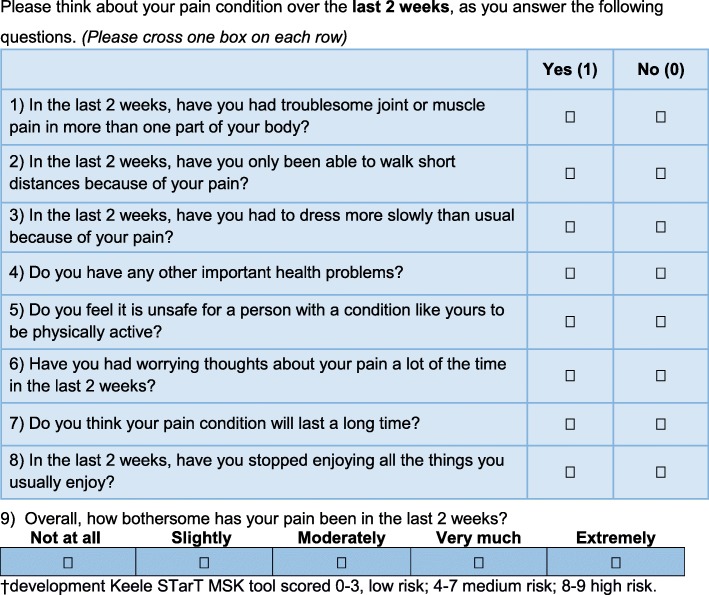
Development version of the Keele STarT MSK Tool© Keele University

**Fig. 2:**
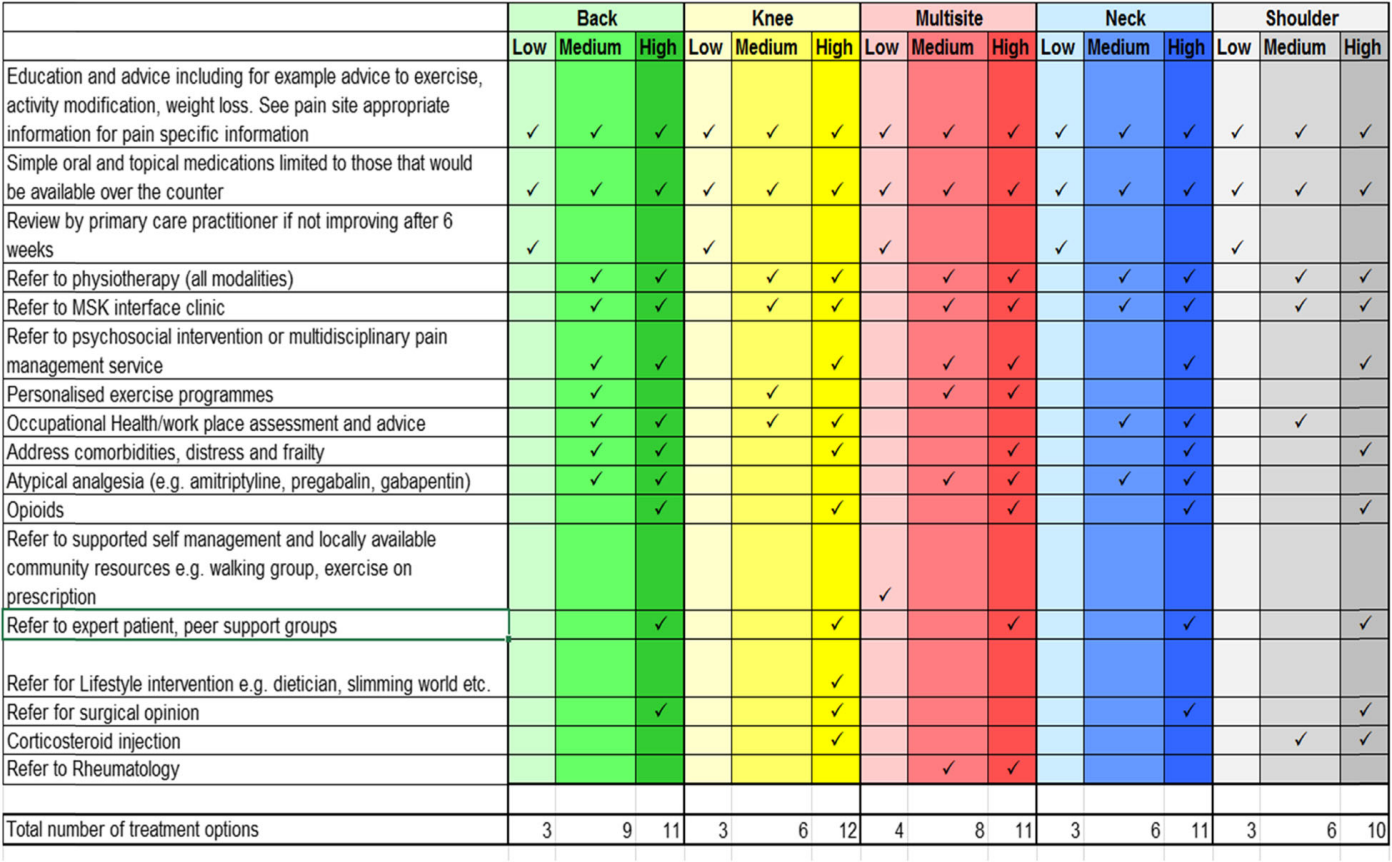
STarT MSK pilot trial recommended matched treatment options

The intervention development was based on the Medical Research Council’s (MRC) framework for the design and evaluation of complex interventions [46]. To support GPs in intervention practices to deliver stratified care, we extended the trial point-of-consultation identification EMR template to also contain the prognostic stratification tool (a development version of the Keele STarT MSK tool) - see Fig. 1 and recommended matched treatment options. The tool was developed and validated in UK General Practice to predict persistent pain and disability and allocate individuals into low, medium or high risk subgroups and is published elsewhere [38]. The recommended matched treatment options for each subgroup are provided in Fig. 2 and were developed through a systematic review and expert consensus process, described in detail elsewhere [39, 40]. In brief, for patients at low risk the treatment options were restricted to supporting self-management and over-the-counter medication, discouraging unnecessary investigations or referral. For those at medium risk, they included referral to conservative non-pharmacological treatments (e.g. those offered by physiotherapists) and workplace assessment and advice, and for those at high risk, they included referral for corticosteroid injections specialist clinical services (including rheumatology, orthopaedics and pain clinics), and opioids.

GP training (3–4 h) within intervention practices was facilitated by an experienced GP trainer (VC) and the lead author (JH) and included: the rationale for stratified care, how it differs from usual care, familiarisation with the EMR template and its fit within the consultation, as well as addressing any questions or concerns. GPs also received a training-update half-way through their recruitment period at which feedback data were shared about individual GP intervention fidelity, with peer-to-peer comparisons and discussion.

### Outcomes measures and analyses

The defined pre-specified measures and success criterion to address each pilot trial objective were as below, with no changes once the pilot commenced:

#### Objective 1

To examine the recruitment and retention rates of general practices we examined the numbers of expressions of interest, face-to-face introductory meetings and signed agreements to participate. To examine the recruitment and retention rates for individual participants we examined the numbers of: participant identification screen activations in the EMR (these were potentially eligible patients screened by the GP at the point-of-consultation) and completions (confirmed eligibility and therefore invited by post to participate), as well as the initial questionnaires returned with written consent to participate in data collection, and monthly and 6-month questionnaires returned. Questionnaire items were examined to identify missing items and any floor-or-ceiling effects. Means and/or medians, standard deviations were reported for all the participant self-reported measures.

The pre-specified success criteria for this objective was that the trial participant identification screen would be activated in approximately 2000 consultations leading to a minimum of 500 participants participating in data collection within an expected 3-month recruitment period and a follow-up rate of > 75% with less than 5% missing items in participant questionnaires.

#### Objective 2

To examine evidence of recruitment selection bias we descriptively analysed (means and standard deviations (SD)) the characteristics of intervention and control arm participants, and characteristics of trial participants and non-participants, using information from the EMR participant identification screen at the point-of-consultation (i.e. MSK pain location, pain intensity, age, sex and deprivation score) and within the participant self-reported initial questionnaire (demographic and clinical characteristics, as listed in Additional file 1). The pre-specified success criteria for this objective was to find little evidence of recruitment selection bias either between intervention and control participants, and between study participants and non-participants.

#### Objective 3

To assess GP fidelity to the stratified care intervention at the point-of-consultation we examined the proportion of eligible cases in which GPs used the stratification tool and choose at least one of the recommended matched treatments. Per protocol matched treatments for each subgroup were defined as follows:

- Low risk: must only have low risk treatment options reported in the EMR
- Medium risk: must have at least one medium risk treatment option and none of the high risk options reported in the EMR
- High risk: patients must have reported within the EMR, at least one high risk treatment option, or a referral to an MSK service providing a medium risk treatment option (e.g. physiotherapy or psychological intervention) with tool subgroup information within their referral so that services were aware that an onward referral to a high risk treatment option might be required.

The pre-specified success criteria for this objective were that within relevant MSK pain consultations intervention GPs would:

1. Complete the prognostic stratification tool in:

- > 50% of cases: “Complete success” (proceed to main trial without amendments)
- 40–50% of cases: “Partial success” (proceed to main trial with amendments)
- < 40% of cases: “Unsuccessful” (consider whether or not to proceed to main trial)
  2. Adhere to per protocol matched treatment options in:
- > 65% of cases: “Complete success” (proceed to main trial without amendments)
- 50–65% of cases: “Partial success” (proceed to main trial with amendments)
- < 50% of cases: “Unsuccessful” (consider whether or not to proceed to main trial)

#### Objective 4

To examine differences in GP decision-making and patient self-reported outcomes at the level of intervention and control we conducted secondary descriptive statistical analyses using the anonymised 6-month EMR audit and follow-up questionnaire data. As this was a feasibility and pilot trial the objective was not hypothesis testing of process/health outcomes, there were no pre-specified success criteria and only complete cases were analysed.

There were four sources of data:

1. **The GP EMR participant identification screen** collected identical point-of-consultation data in all 8 GP practices, including the primary MSK pain site/location and average pain intensity (intended primary outcome for the main trial) by asking:

- *How intense was your pain, on average, over the last 2 weeks? [Responses on a 0–10 scale, where 0 is “no pain” and 10 is “worst pain ever”].*

Pain intensity was chosen as the potential primary outcome for the future main trial as it had the strongest face validity with patients during a pre-pilot Patient and Public Involvement and Engagement (PPIE) workshop and is also a recommended outcome for trials testing treatments for MSK pain [47, 48]. In the intervention practices the EMR participant identification screen was extended to embed the stratified care intervention and collect additional data relating to stratification tool item responses and the matched treatment options chosen at the point-of-consultation. All template responses were date stamped and linked to an individual GP and patient. It was also possible from the EMR screen to collect automated data on the MSK consulter’s age, sex and English Index of Multiple Deprivation (IMD) 2015 [49], with non-participants data anonymised first.

2. **Baseline and 6-month postal questionnaires** included self-reported measures for average pain intensity over the last 2 weeks (identical wording and responses to the trial identification template), physical function measures for each of the MSK pain sites (filtered according to GP designation) including the back specific Roland-Morris Disability Questionnaire (RMDQ) [50], the Neck Disability Index (NDI) [51, 52] the Shoulder Pain And Disability Index (SPADI) [53], the Knee Injury and Osteoarthritis Outcome Score Physical Function Short-form (KOOS-PS) [54] and for multi-site pain, the Short Form 12 (v2) Physical Component Scale [55]. Other outcomes were MSK risk status using the development version of the Keele STarT MSK tool [38], overall MSK health status using the Musculoskeletal Health Questionnaire [56], fear avoidance beliefs using the 11-item Tampa Scale of Kinesiophobia [57], patient perceived reassurance (from their GP) using the Effective Consultation and Reassurance Questionnaire (ECRQ) [58] (which has four subscales: information gathering, relationship building, generic reassurance and cognitive reassurance), health-related quality of life using the EuroQol five-dimension, five-level version (EQ-5D-5 L) [59], single items each capturing satisfaction with care received, whether participants had received written education material from their GP about their MSK problem (yes/no), and overall rating of global change (− 5 to + 5 numerical response scale) since their index GP visit (the one in which the trial EMR screen was activated and they were invited to participate in the study data collection) [60], whether they were in paid employment and had taken any work absence due to their MSK pain, and an item asking how their productivity at work is affected (0–10 NRS). Patient population descriptors (captured at baseline alone) included; the Single Item Health Literacy Screener (SILS) [61] and pain episode duration by asking “how long is it since you had a whole month without [insert pain site e.g. back] pain”. Additional file 1 provides a summary of the self-reported measures collected.
3. **Monthly follow-up**

Three items were collected using monthly follow-up via Short Message System (SMS) text or one-page postal questionnaire (depending on participant preference): average pain intensity (same wording as GP EMR screen), distress due to pain, and pain self-efficacy using:

- *How much distress have you been experiencing because of your pain, on average, over the last 2 weeks? [Responses from 0 = no distress to 10 = extreme distress]*
- *How confident have you felt about managing your pain by yourself* e.g. *medication, changing lifestyle? [Responses from 0 = not at all confident to 10 = extremely confident]*
  4. **Anonymised GP medical record audit**

An anonymised audit of medical record data from all 8 GP practices for patients in whom the trial EMR participant identification screen had been completed, including:

i) **prescriptions** (categorised into simple analgesics, non-steroidal anti-inflammatories (NSAIDs), neuromodulators, muscle relaxants, corticosteroid injections and opioids)
ii) **referrals** (categorised into physiotherapy/MSK interface services, secondary care specialist services including orthopaedics, pain clinics, and rheumatology)
iii) **imaging** (categorised into x-rays/MRI scans, MSK ultrasound scans and bone density scans)
iv) **sick certifications or ‘fit-notes’** (categorised into number per patient and mean length in days)
v) **repeat MSK general practice consultations.**

### Sample size

Whilst sample size calculations for pilot cluster trials are known to be difficult [62], the initial plan was to carry out an internal pilot trial with a 3-month recruitment phase, that mirrored the methods of the main cluster trial but was limited to assessing feasibility within 8 GP practices (4 intervention and 4 control) prior to involvement of a further 22 GP practices (30 in total). If the internal pilot had achieved its success criteria, we had planned that these 8 randomised practices would continue to recruit patients for a full 6-month period, and their data included in the main trial. Hence, we anticipated recruiting 500 patients from the 8 practices over the first 3-months in the internal pilot trial, with a further 500 participants to be recruited from those practices (and in addition 2750 from a further 22 practices for the main trial phase).

## Results

### Objective 1: general practice and participant recruitment and retention rates

There were 32 general practices who expressed an initial interest in participating in the pilot trial from the West Midlands region of England, of which 16 agreed to a face-to-face introductory meeting with the research team, and 8 were recruited (with written agreements) and randomised (4 intervention, 4 control). The reasons given for declining participation included the practice lacking capacity in terms of resource at that particular time (*n* = 2), unwillingness to participate in the training session (*n* = 2), unwilling to use the EMR participant identification screen (*n* = 2), being already involved with another MSK pain research study (*n* = 1), and a perception that the practice’s patient population would struggle to respond to the self-report questionnaires (*n* = 1). The 8 participating practices had a total adult practice population size of 58,307 (25,697 intervention, 32,610 control). The smallest practice had 3 GPs and a registered adult population of 3992; the largest had 9 GPs and 13,359 adult patients. In total 59 GPs identified patients for the trial (39 in control practices and 20 in intervention practices).

**Fig. 3:**
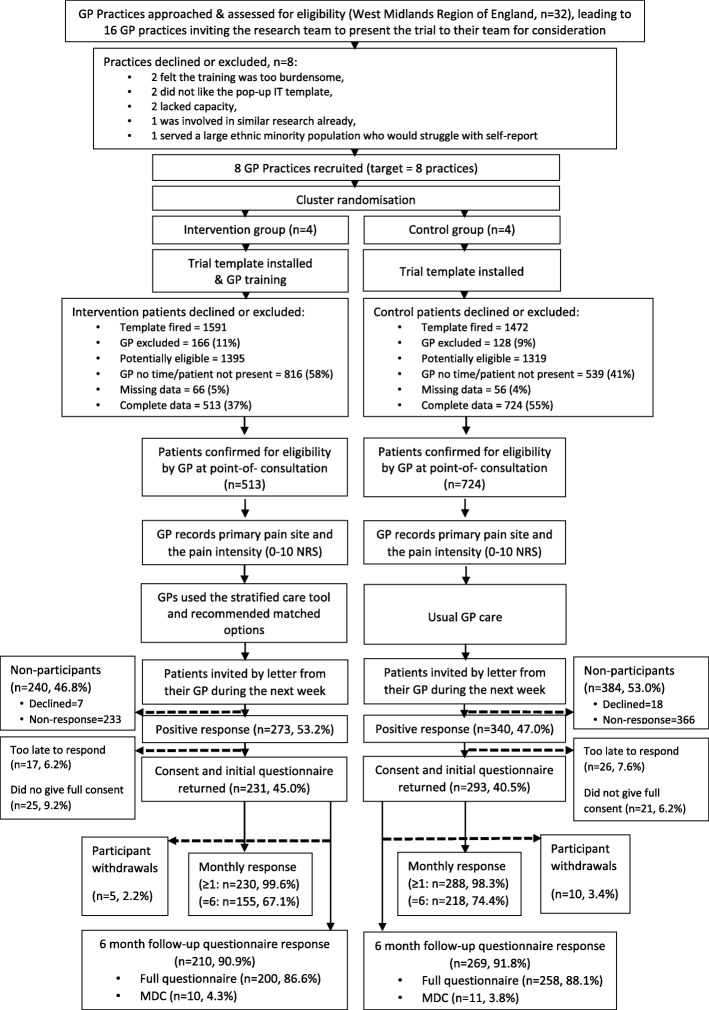
Participant flowchart

Patient recruitment and follow-up through the trial are described in Fig. 3. Recruitment started on 11/10/2016 and the last practice template was deactivated on 24/05/2017 with the last invite reminder sent on 21/06/2017 and last patient provided consent to data collection on 21/07/2017. There were 3063 potentially eligible patients screened by GPs at the point-of-consultation, the EMR participant identification screen was completed in 1281 with confirmed eligibility, of whom 1237 were actually invited by postal letter to participate in data collection, 567 initial questionnaires returned with written consent to participate in data collection, and 524 responses were received within the 4-week eligibility time-period (231 intervention and 293 controls). To recruit 500 patients took 28 weeks, more than twice as long as the original estimate (12 weeks). Recruitment varied substantially between the 8 practices (range *n* = 11–127) suggesting the need to account for this variation within the main trial sample size calculation. Once 500 participants were recruited, the EMR participant identification screen in practices was switched off, however, we recruited a further 24 participants (*n* = 524 in total) over the following month (33 weeks in total) due to the time lag in sending invitations and receiving patient consent to data collection (via the post).

The overall participant 6-month follow-up rate for the intended future RCT primary outcome of pain intensity was 477/524 (91.0%); usual care 209/231 (90.4%), intervention 268/293 (91.4%). Response rates for monthly pain intensity scores at 5 or more time-points (max. Possible was 6) was 82.6%, with data for 3 time-points available in 91.8%. 15 patients withdrew over the 6 months follow-up period: 5 from intervention practices (2 due to illness/surgery/poor health, 1 due to moving house, and 2 did not want further contact about the study), and 10 from control practices (5 due to illness/surgery/poor health, 1 had died (unrelated), 2 withdrew because they felt recovered, and 2 did not want further contact). There were no related, unexpected serious adverse events or harms reported. At 6-month follow-up patients reported 11 hospital admissions (5 intervention, 6 control) related to their MSK pain (e.g. knee replacement or shoulder surgery). Missing data items in the questionnaires remained less than 5%. Anonymised medical record data were available for 1281 patients (529 from intervention practices and 752 from control practices).

The success criteria for this objective (*the template activated in approximately 2000 consultations leading to a minimum of 500 participants providing consent within an expected 3-month recruitment period and a follow-up rate of > 75% with less than 5% missing items in patient questionnaires*) was only “partially successful”, as although patient recruitment and retention were “successful”, the timeline needed to recruit 500 patients was 28 rather than 12 weeks.

The learning/change needed ahead of the main trial included reducing the main trial sample size (following discussion with the independent Trial Steering Committee and funder) by removing the pre-specified sub-group analysis (at the risk-subgroup level) and instead powering the trial for the overall comparison between intervention and control arms. In addition, the main trial sample size was re-calculated based on the following: Firstly, the pilot recruitment rate showed that the template was completed in just under 40% of cases, and from the subsequent letter of invitation 40% returned their initial questionnaire and provided consent to participation in the data collection (on average, 60 patients per practice). A conservative estimate (50 patients per practice) was therefore used for the main trial. Secondly, the proportions expected within each of the three risk subgroups, as determined from the self-complete questionnaires, were revised based on the pilot trial findings, to: 32% low risk, 55% medium risk, 13% high risk. This was important as the trial was powered to detect superiority of stratified care in the medium and high risk subgroups, with an expected effect size of 0.20.

Thirdly, for GP cluster parameterisation, we made the following estimates, based primarily on previous guidelines, as pilot trial figures need to be viewed cautiously given the possible lack of precision [62]. For the main trial primary outcome (pain intensity) we have conservatively allowed for an intracluster correlation coefficient (ICC) of 0.01 based on a guideline from previous primary care trials [63] and the pilot trial ICC being considerably lower (0.004). Our main trial estimated coefficient of variation in recruitment per practice is also based on a guideline estimate of 0.65 [64] as well as the pilot being similar at 0.66. Our expected loss to follow-up across all time-points is conservatively estimated at 25%, which in the pilot was around 5%. Lastly, our repeated measures correlation is estimated using a guideline figure of 0.7 [65], which is conservative based on our pilot trial figure of 0.65. These factors combine to give a sample size inflation factor of × 2.3 (based on an average cluster size of about 50 participants per practice in 6 months). Correlation of data within 6 repeated measurements and correlation of follow-up scores with baseline score are typically 0.7 and 0.5, respectively which combine to give a sample size deflation factor of × 0.5). The product of inflation and deflation effects result in a magnification of 1.15 compared to a conventional, individual-patient, single follow-up comparison, whereby the sample size requirement would be 525 per treatment arm (or, 1050 in total). The adjusted sample size target for the main trial was is therefore 600 patients per arm (1200 in total) from approx. 24 general practices (approx. 12 per arm).

### Objective 2: to examine evidence of selection bias

Table 1 shows a descriptive evaluation of individual participant demographics and characteristics (split by trial arm) and participants and non-participants. Whilst most characteristics were similar (e.g. sex) between intervention and control arms suggesting minimal selection bias, there were a few differences between participants (e.g. overall, they were slightly older and from more deprived areas) and non-participants. Mean pain intensity (0–10 Numerical Response Scale (NRS)) at the point-of-consultation was similar between participants (6.33, SD 2.05) and non-participants (6.35, SD 2.10), but pain scores were 0.5 points higher in participants in the intervention arm than control, although this difference had disappeared by the time of the initial patient questionnaire (typically 1–3 weeks later).

**Table 1:** Patient baseline characteristics

Key characteristics	All participants (*n* = 524)	Intervention participants (*n* = 231)	Control participants (*n* = 293)	Non participants (*n* = 713^a^)
Age, mean (SD)	61.1 (14.8)	60.3 (15.1)	61.8 (14.5)	53.8 (17.8)
Female, n (%)	318 (60.7%)	133 (57.6%)	185 (63.1%)	416 (58.4%)
Index Multiple Deprivation quintile, n (%)				
1 (least deprived)	8 (1.5%)	7 (3.0%)	1 (0.3%)	11 (1.6%)
2	55 (10.6%)	17 (7.4%)	38 (13.0%)	102 (14.4%)
3	104 (19.9%)	55 (23.9%)	49 (16.7%)	152 (21.4%)
4	143 (27.3%)	51 (22.2%)	92 (21.4%)	230 (32.4%)
5 (most deprived)	213 (40.8%)	100 (43.5%)	113 (38.6%)	216 (30.4%)
GP Practice, n (%)				
A	49 (9.4%)	49 (21.2%)	–	84 (11.8%)
B	11 (2.1%)	–	11 (3.8%)	17 (2.4%)
C	121 (23.1%)	–	121 (41.3%)	197 (27.6%)
D	30 (5.7%)	30 (13.0%)	–	23 (3.2%)
E	59 (11.3%)	59 (25.5%)	–	76 (10.7%)
F	93 (17.8%)	93 (40.3%)	–	99 (13.9%)
G	127 (24.2%)	–	127 (43.3%)	168 (23.6%)
H	34 (6.5%)	–	34 (11.6%)	49 (6.9%)
Pain location, n (%)				
Knee	144 (27.5%)	62 (26.8%)	82 (28.0%)	–
Neck	59 (11.3%)	30 (13.0%)	29 (9.9%)	–
Back	155 (29.6%)	73 (31.6%)	82 (28.0%)	–
Shoulder	124 (23.7%)	53 (22.9%)	71 (24.2%)	–
Widespread pain	42 (8.0%)	13 (5.6%)	29 (9.9%)	–
Duration (time since whole month without pain), n (%)				
< 3 months	136 (26.0%)	69 (29.9%)	67 (22.9%)	–
3–6 months	77 (14.7%)	32 (13.9%)	45 (15.4%)	–
7–12 months	89 (17.0%)	38 (16.5%)	51 (17.4%)	–
1–2 years	75 (14.3%)	30 (13.0%)	45 (15.4%)	–
3–5 years	53 (10.1%)	21 (9.1%)	32 (10.9%)	–
6–10 years	48 (9.2%)	20 (8.7%)	28 (9.6%)	–
> 10 years	46 (8.8%)	21 (9.1%)	25 (8.5%)	–
Health Literacy Single Item Screen (Need help), n (%) [*n* = 516]				
Never/rarely/sometimes	500 (96.9%)	222 (98.3%)	278 (95.9%)	–
Often/always	16 (3.2%)	4 (1.8%)	12 (4.2%)	–
Comorbidities (No. of listed long-term conditions), n (%)				–
0	186 (35.5%)	86 (37.2%)	100 (34.1%)	–
1	161 (30.7%)	79 (34.2%)	82 (28.0%)	–
2	130 (24.8%)	52 (22.5%)	78 (26.6%)	–
≥ 3	47 (9.0%)	14 (6.1%)	33 (11.3%)	–
Lives alone (Yes), n (%) [*n* = 523]	87 (16.6%)	40 (17.3%)	47 (16.1%)	–
Currently employed (Yes), n (%) [*n* = 509]	234 (46.0%)	104 (46.6%)	130 (45.5%)	–
Pain interference with performance at work (0–10, the higher score the worse), mean (SD)	4.28 (3.06) [*n* = 257]	3.87 (2.88) [*n* = 113]	4.60 (3.16) [*n* = 144]	–
Time-off-work last 6 m due to MSK pain, n (%) [*n* = 260]	66 (25.4%)	28 (24.8%)	38 (25.9%)	–
Receipt of written information from GP, n (%) [*n* = 520]	213 (41.0%)	163 (71.5%)	50 (17.1%)	–
Pain intensity (at the point of GP consultation) (0–10, the higher score the worse), mean (SD)	6.33 (2.05)	6.60 (1.93)	6.11 (2.11)	6.35 (2.10)^‡^
Pain intensity (self-reported in baseline questionnaire) (0–10, the higher score the worse), mean (SD)	6.21 (2.25) [*n* = 523]	6.22 (2.17) [*n* = 230]	6.21 (2.32) [*n* = 293]	–
Self-efficacy (confidence to manage MSK pain) (0–10, the higher score the better), mean (SD)	5.43 (2.62) [*n* = 521]	5.41 (2.67) [*n* = 228]	5.44 (2.59) [*n* = 293]	–
Distress (0–10, the higher score the worse), mean (SD)	5.66 (2.61) [*n* = 524]	5.62 (2.60) [*n* = 231]	5.69 (2.61) [*n* = 293]	–
Days of moderate physical activity per week, median (IQR)	2 (0–4) [*n* = 521]	2 (0–4) [n = 230]	2 (0–4) [*n* = 291]	–
No. of previous MSK pain episodes, median (IQR)	5 (1–25) [*n* = 415]	5 (1–15) [*n* = 186]	5 (1–30) [*n* = 229]	–
MSK Risk status (Keele development version of the STarT MSK Tool – note it was not the final version), mean (SD) [*n* = 482]				–
Low risk (0–3 score), n (%)	155 (32.2%)	67 (30.9%)	88 (33.2%)	–
Medium risk (4–7 score), n (%)	263 (54.6%)	119 (54.8%)	144 (54.3%)	–
High risk (8–9 score), n (%)	64 (13.3%)	31 (14.3%)	33 (12.5%)	–
Overall musculoskeletal health status (MSK-HQ) (0–56, the higher score the better), mean (SD)	29.6 (10.4) [*n* = 507]	29.9 (10.5) [*n* = 223]	29.4 (10.4) [*n* = 284]	–
Overall global change (−5–5, the higher score the better), mean (SD)	0.34 (2.08) [*n* = 523]	0.41 (2.19) [*n* = 230]	0.28 (1.99) [*n* = 293]	–
Fear-avoidance (using 11-item TSK, higher score the worse) mean (SD)	24.5 (6.80) [*n* = 511]	24.3 (6.60) [*n* = 224]	24.7 (6.94) [*n* = 287]	–
Satisfaction with initial GP care [*n* = 522]				
Very satisfied, n (%)	140 (26.8%)	67 (29.1%)	73 (25.0%)	–
Quite satisfied, n (%)	184 (35.3%)	81 (35.2%)	103 (35.3%)	–
No opinion, n (%)	115 (22.0%)	43 (18.7%)	72 (24.7%)	–
Not very satisfied, n (%)	74 (14.2%)	34 (14.8%)	40 (13.7%)	–
Not at all satisfied, n (%)	9 (1.7%)	5 (2.2%)	4 (1.4%)	–
Patient perceived reassurance from GP for MSK pain (higher score is better)				
Data gathering, mean (SD)	9.9 (4.3) [*n* = 502]	10.5 (4.6) [*n* = 223]	9.5 (4.1) [*n* = 279]	–
Relationship building, mean (SD)	11.6 (4.2) [*n* = 499]	12.0 (4.4) [*n* = 220]	11.3 (3.9) [*n* = 279]	–
Generic, mean (SD)	13.1 (4.7) [n = 507]	13.2 (5.0) [n = 224]	13.0 (4.5) [*n* = 283]	–
Cognitive, mean (SD)	13.4 (4.7) [*n* = 510]	13.5 (4.9) [*n* = 223]	13.2 (4.6) [*n* = 287]	–
Total, mean (SD)	48.0 (16.0) [*n* = 510]	49.2 (17.2) [*n* = 224]	47.1 (15.0) [*n* = 286]	–
Knee physical function using KOOS (the higher score the better), mean (SD)	42.9 (21.2) [*n* = 142]	44.0 (22.1) [*n* = 61]	42.0 (20.5) [*n* = 81]	–
Neck physical function using NDI (the higher score the worse), mean (SD)	16.1 (8.02) [*n* = 59]	14.6 (6.39) [*n* = 30]	17.7 (9.28) [*n* = 29]	–
Back physical function using RMDQ (the higher score the worse), mean (SD)	9.59 (5.50) [*n* = 155]	9.84 (5.40) [*n* = 73]	9.38 (5.57) [*n* = 82]	–
Shoulder function using SPADI-Function (the higher score the worse), mean (SD)	47.1 (24.8) [*n* = 124]	45.9 (25.3) [*n* = 53]	48.0 (24.5) [*n* = 71]	–
Multi-site physical function using SF12 PCS the higher score the better), mean (SD)	34.4 (9.52) [*n* = 42]	35.5 (9.35) [*n* = 13]	33.9 (9.72) [*n* = 29]	–
Quality of life using EQ5D-5 L, mean (SD)	0.56 (0.24) [*n* = 513]	0.55 (0.25) [*n* = 226]	0.57 (0.22) [*n* = 287]	–

^a^ 43 patients were excluded as they returned their baseline questionnaire after 28 days (17 intervention arm; 26 control arm); 80 baseline responders did not give full consent to study (39 intervention arm; 41 control arm). Too late and non-consent figures were not mutually exclusive: 9 patients were late and did not consent to study (3 intervention arm; 6 control arm). Hence, 114 patients were excluded for either lateness or non-consent (53 in intervention arm; 61 in control arm); 599 patients did not respond (229 in intervention arm; 370 in control arm). ^‡^ Those in whom the trial template was completed at the point of consultation, including participants and non-participants

Overall there were few differences across other characteristics and the pre-specified success criteria for this objective of finding little evidence of selection bias was judged “successful”. There were, therefore, no changes required to recruitment procedures for the main trial.

### Objective 3: assessing GP fidelity to the stratified care intervention

GPs from intervention practices used the stratification tool within the EMR in 513/1591 (32%) of eligible patients, which was “unsuccessful” according to our pre-specified success criteria. GP fidelity to choosing recommended matched treatment options (shown in Table 2) achieved “complete success” with 81% of patients at low risk, 89% for medium risk and 87% for patients at high risk being correctly matched to a recommended treatment.

**Table 2:** GP fidelity to the recommended matched treatment options

Matched GP treatment options	Low risk (*n* = 161, 38%)		Med risk (*n* = 224, 52%)		High risk (*n* = 45, 10%)		Grand Total
Advice - verbal	102	63%	108	48%	23	51%	233
Advice - written	91	57%	140	63%	17	38%	248
Advice – over-the-counter medication	84	52%	10	4%			94
Advise GP follow-up if symptoms persist	66	41%	12	5%			78
Refer to Physiotherapy	2	2%	85	84%	14	14%	101
Refer to MSK interface clinic			38	17%	10	22%	48
Refer to pain clinic (multi-disciplinary)			1	0%	3	7%	4
Personalised exercise programme			5	2%	1	2%	6
Refer to Occupational Health support			15	7%	3	7%	18
GP address comorbidity, distress or frailty	1	1%	7	3%	7	16%	15
Prescribe atypical analgesia	2	1%	59	26%	9	20%	70
Prescribe opioids			1	0%	10	22%	11
Signpost to peer support group					2	4%	2
Signpost/refer to lifestyle interventions					2	4%	2
Refer for surgical opinion	3	2%	4	2%	7	16%	14
Corticosteroid injection	1	1%			4	9%	5
Refer to rheumatology			2	1%	1	2%	3
Fidelity to stratified care in decision-making		Pt count		%			
Low risk - per protocol		130		81%			
Medium risk - per protocol		200		89%			
High risk - per protocol		39		87%			
Low risk - given Medium treatments		3		2%			
Low risk - given High treatments		3		2%			
Medium risk – given Low treatments		0		0%			
Medium risk - given High treatment		5		2%			
High risk – given Low treatments only		3		7%			
High risk – given Medium treatments		0		0%			
Low risk – only tool used (no treatments selected)		25		16%			
Med risk – only tool used (no treatments selected)		19		8%			
High risk – only tool used (no treatments selected)		3		7%			
Grand Total		430					

Through the nested qualitative research (reported separately, [41]) and feedback discussions with the participating GPs about the reasons for the low rate of completion of the tool, we gathered a number of insights to inform the main trial. Firstly, GPs perceived that the using the whole EMR template increased their consultation workload and asked for the treatment options to be simplified. They also reported that the stratified care intervention was only appropriate for consultations where MSK pain was the primary reason for the consultation, where they could focus on the MSK pain problem. GPs also admitted that patients had frequently left the consultation room before they used the EMR and that they did not use the tool when their clinics were very busy. We therefore agreed in the future main trial to lower the expected proportion of MSK related consultations in which the tool would be used at the point-of-consultation from 50 to 25%. We also identified that some GPs rarely coded MSK pain consultations and that others tended to use ‘Synonym’ codes, which are set of diagnostic codes that needed to be removed from the list of codes used to activate the EMR participant identification screen, as they caused it to activate in error for a range of non-MSK pain problems (e.g. chest pain). It was agreed that for the main trial the GP training needed to include ways to mitigate these issues. GPs also recommended reducing the 4 h of intervention training to 2 h and to provide a dedicated NHS physiotherapy pathway for patients in the main trial to overcome GPs’ concerns about over-loading physiotherapy services with patients with MSK pain. Finally, GPs reported feeling uncomfortable with the self-report style wording of the development version of Keele STarT MSK tool. For example, they felt certain items could be modified to be less ‘clunky and awkward’ to ask (e.g. item 4: “Do you have any other important health problems?” which confused/unsettled patients when asked by their own family doctor who they expected to know their health problems well). We therefore developed a clinician-completed version of the Keele STarT MSK tool for use in the main trial, to overcome these wording problems, but keeping the item constructs as similar as possible. A license to obtain both the original self-report and clinician completed versions of the tool is available on request at www.keele.ac.uk/startmsk.

### Objective 4: describing GP decision-making and patient outcomes in both arms

The results from the EMR audit of GP decision-making in MSK consultations are shown in Table 3 (split by intervention and control). GPs in intervention practices prescribed less opioids and more over-the-counter medication and anti-inflammatories than GPs in control practices. In addition, they gave more written self-management information to patients, used less MSK-related imaging and referred patients to physiotherapy earlier than in control practices. Numbers of corticosteroid injections, sick certifications, and repeat MSK pain related general practice consultations over 6 months were similar in intervention and control practices.

**Table 3:**
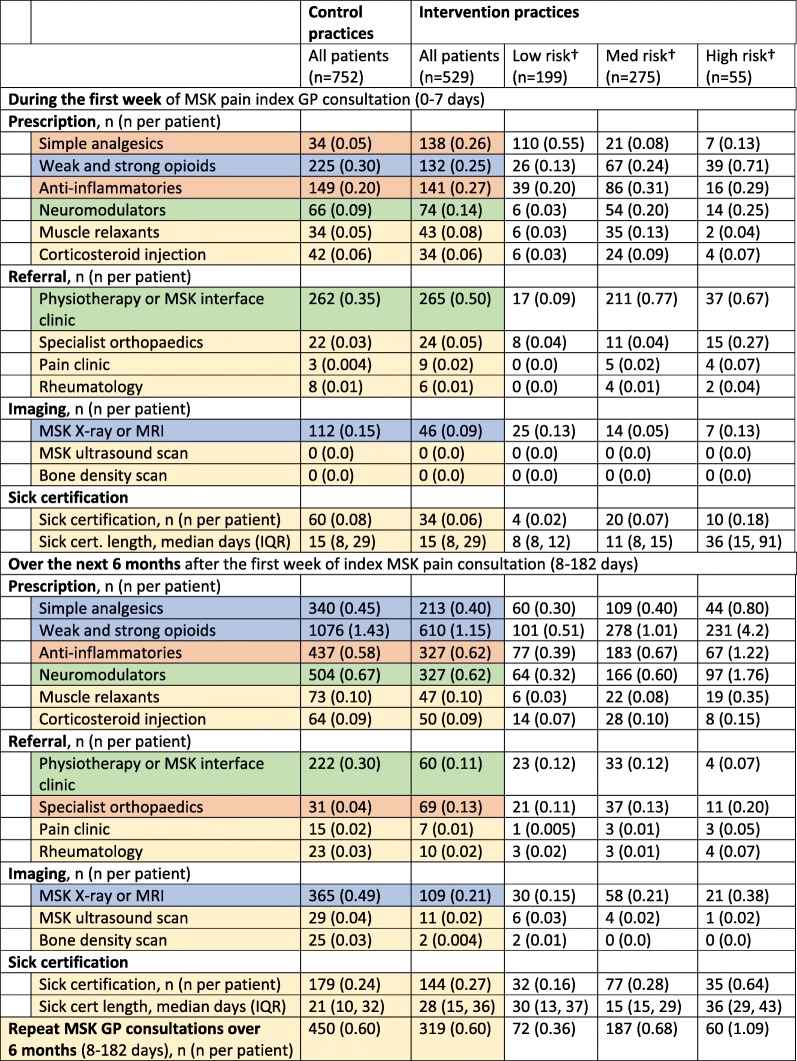
Comparison of GP decision-making between intervention and control practices.

†STarT MSK scored 0–3, low risk; 4–7 medium risk; 8–9 high risk.

The colours represent the effects of the intervention on GP behaviours in comparison to controls:

Reduced (> 0.04)  Same  Increased (> 0.04)  Provided earlier .

“It should be noted that the numbers of patients referred for an x-ray or MRI are combined, as in both the intervention and control GP practices, MRI was used less than 5 times in total, which meant there were too few numbers for any meaningful comparison of MRI alone.”

Descriptive data on patients’ clinical outcomes over 6-months follow-up are presented in Table 4. Mean (SD) 6-month pain intensity was 3.93 (2.98) in participants in intervention practices and 4.18 (2.88) in control. Most other 6-month outcomes were similar although there was less MSK-related time-off-work in participants from intervention (17.4%) than control practices (25.4%). We did not statistically compare these outcomes in this pilot trial.

**Table 4:** Clinical outcome measures at 6-month follow-up by intervention arm

Key characteristics	Intervention 6 m follow-up (*n* = 200)	Control 6 m follow-up (*n* = 258)
6-month pain intensity (self-reported), mean (SD)	3.93 (2.98) [*n* = 209]^a^	4.18 (2.88) [*n* = 268]^a^
Change in pain intensity (0–10, higher score is worse), from GP consultation to 6-month Questionnaire, mean (SD)	−2.6 (3.1) [*n* = 207]	−1.9 (3.1) [*n* = 266]
Pain interference with performance at work (0–10, the higher score the worse), mean (SD)	3.14 (2.74) [*n* = 87]	3.86 (3.13) [*n* = 115]
Days of moderate physical activity per week, median (IQR)	3 (1–4) [*n* = 199]	3 (1–4) [n = 257]
Currently employed (Yes), n (%)	78 (40.2%)	101 (39.9%)
Time-off-work last 6 m due to MSK pain (Yes), n (%)	15 (17.4%)	29 (25.4%)
Overall global change (−5–5, the higher score the better), mean (SD)	1.20 (2.72) [*n* = 199]	1.15 (2.62) [*n* = 257]
Risk status using a development version of the Keele STarT MSK Tool, mean (SD) (note: not the final version)	3.40 (2.70) [*n* = 190]	3.64 (2.35) [*n* = 234]
Low risk (0–3 score), n (%)	113 (59.5%)	127 (54.3%)
Medium risk (4–7 score), n (%)	60 (31.6%)	93 (39.7%)
High risk (8–9 score), n (%)	17 (9.0%)	14 (6.0%)
Overall musculoskeletal health status (MSK-HQ, 0–56, the higher score the better), mean (SD)	37.5 (12.8) [*n* = 193]	37.3 (11.8) [*n* = 248]
Fear-avoidance (using 11-item TSK, higher score the worse) mean (SD)	22.81 (7.25) [*n* = 197]	23.70 (7.24) [*n* = 253]
Satisfaction with GP care for MSK pain		
Very satisfied, n (%)	48 (24.2%)	58 (22.8%)
Quite satisfied, n (%)	71 (35.9%)	89 (34.9%)
No opinion, n (%)	46 (23.2%)	60 (23.5%)
Not very satisfied, n (%)	25 (12.6%)	44 (17.3%)
Not at all satisfied, n (%)	8 (4.0%)	4 (1.6%)
Knee physical function using KOOS (the higher score the better), mean (SD)	51.7 (24.5) [*n* = 55]	53.6 (22.9) [*n* = 72]
Neck physical function using NDI (the higher score the worse), mean (SD)	7.80 (5.83) [*n* = 27]	11.89 (11.57) [*n* = 24]
Back physical function using RMDQ (the higher score the worse), mean (SD)	6.90 (6.52) [n = 61]	6.44 (5.80) [*n* = 75]
Shoulder physical function using SPADI-Function (the higher score the worse), mean (SD)	30.2 (29.6) [*n* = 44]	33.4 (27.8) [*n* = 62]
Multi-site physical function using SF12 PCS the higher score the better), mean (SD)	37.3 (15.1) [*n* = 12]	34.7 (10.7) [n = 23]
Last 6 months saw a professional for MSK pain [*n* = 421]	126 (67.0%)	175 (75.1%)
Last 6 months received any MSK investigation/treatment [*n* = 412]	66 (36.7%)	66 (28.5%)
Last 6 months had MSK hospital overnight stay [*n* = 446]	5 (2.6%)	6 (2.4%)
Quality of life using EQ5D-5 L, mean (SD)	0.65 (0.26) [*n* = 208]^a^	0.63 (0.25) [*n* = 258]^a^

^a^ Additionally includes minimal data collection (MDC) responses hence the denominator numbers (n) are greater than the total column numbers of 200 (intervention) and 258 (control) which reflect total questionnaire returns

## Discussion

This feasibility and pilot trial examined the feasibility of a future definitive cluster RCT in respect to recruitment and retention rates, potential selection bias and GP intervention fidelity to stratified care at the point-of-consultation for adults with MSK pain.

Our original plan was that this study was an internal feasibility and pilot trial. Our findings showed that participant retention rates were high, that GPs matched patients to recommended treatment options well (> 80% of cases), and there was little evidence of selection bias, therefore the cluster trial design was deemed suitable for the future main trial. However, the length of time taken to recruit participants was over twice as long as expected (28 rather than 12 weeks), and GPs completed the Keele STarT MSK Tool in fewer patient cases than we had hoped for (they used it in 32% of patient cases when the target was > 50%). The nested qualitative study findings [41] and feedback discussions with participating GPs explored the reasons why only two of the four pre-specified pilot trial success criteria were met. These identified in the particular challenge of using the EMR template and stratified care intervention when MSK pain was not the primary reason for the consultation.

GPs also suggested a number of positive changes to make prior to the future definitive RCT and thus this study became an external pilot trial. These changes included simplifying the recommended treatment options and developing a clinician-completed version of the Keele STarT MSK Tool. Furthermore, we agreed to lower the expected proportion of MSK consultations in whom the tool would be used from 50 to 25% as we were unable to stop the EMR template from firing in consultations where MSK pain was a multimorbidity and not the main focus of the consultation. We also agreed to give GPs training specifically about the issue with ‘Synonym’ codes that failed to activate the EMR participant identification screen and reduced the intervention GP training from 4 h to 2 h. Lastly, we organised for NHS physiotherapy services receiving patients from participating intervention practices to provide a dedicated pathway for patients in the main trial. This pathway was put in place to overcome GPs’ concerns about their referrals over-loading NHS physiotherapy services with patients with MSK pain and we specified that is was strictly not allowed to increase the speed of access to physiotherapy treatment for intervention participants.

The main STarT MSK trial is currently ongoing (ISRCTN15366334).

## Conclusions

This feasibility and pilot trial has successfully demonstrated the feasibility of the cluster RCT design with high retention rates over 6 months (> 90%) and little evidence of selection bias, although changes to the main trial sample size were required due to a slower than expected recruitment rate. GP point-of-consultation fidelity to the stratified care intervention was mixed with GPs using the tool less often than expected (only when they coded consultations, when they had time and when MSK pain was the primary reason for the visit). However, there was high fidelity to choosing recommended matched treatment options (> 80% of cases). The learning from this feasibility and pilot RCT has led to a number of important changes prior to the main STarT MSK trial testing the clinical and cost-effectiveness of stratified primary care for patients with MSK pain.

## Supplementary information

**Additional file 1.** Summary of participant self-reported measures.

## Data Availability

In line with the Standard Operating Procedures in place at the Clinical Trials Unit (CTU) at Keele University, and data are archived at a dedicated location within the Keele CTU network. A request to access archived data can be made by completion of a Data Transfer Request form, which can be accessed by contacting the Keele CTU at the David Weatherall Building, Keele University, Staffordshire, ST5 5BG; Tel: + 44 (0) 1782 733905.

## Abbreviations

CTU: Clinical trials unit
DALYs: Disability adjusted life years
ECRQ: Effective consultation and reassurance questionnaire
EMR: Electronic medical record
EQ-5D-5 L: EuroQol five-dimension, five-level version
GP: General practitioners
IMD: Index of multiple deprivation
ISRCTN: International standard randomised controlled trials number
KOOS-PS: Knee injury and osteoarthritis outcome score physical function short-form
MRC: Medical research council’s
MSK: Musculoskeletal
NDI: Neck disability index
NIHR: National institute for health research
NIHR CRN: National institute for health research clinical research network
NRS: Numerical response scale
NSAIDs: Non-steroidal anti-inflammatories
PPIE: Public Involvement and Engagement
RCT: Randomised controlled trial
RMDQ: Roland-morris disability questionnaire
SD: Standard deviation
SILS: Single item health literacy screener
SMS: Short message system
SPADI: Shoulder pain and disability index
TAPs: Treatment of aches and pains

## References

[1] Jordan KP, Kadam UT, Hayward R, Porcheret M, Young C, Croft P (2010). Annual consultation prevalence of regional musculoskeletal problems in primary care: an observational study. BMC Musculoskelet Disord.

[2] Vos T (2015). Global, regional, and national incidence, prevalence, and years lived with disability for 301 acute and chronic diseases and injuries in 188 countries, 1990-2013: a systematic analysis for the global burden of disease study 2013. Lancet..

[3] Collaborators GDaH. Global, regional, and national disability-adjusted life-years (DALYs) for 315 diseases and injuries and healthy life expectancy (HALE), 1990-2015: a systematic analysis for the global burden of disease study 2015. Lancet 2016;388(10053):1603–1658. 10.1016/S0140-6736(16)31460-X PMC5388857 27733283

[4] Barnett K, Mercer SW, Norbury M, Watt G, Wyke S, Guthrie B (2012). Epidemiology of multimorbidity and implications for health care, research, and medical education: a cross-sectional study. Lancet.

[5] Brand CA, Ackerman IN, Tropea J (2014). Chronic disease management: improving care for people with osteoarthritis. Best Pract Res Clin Rheumatol.

[6] Paskins Z, Sanders T, Croft PR, Hassell AB (2015). The identity crisis of osteoarthritis in general practice: a qualitative study using video-stimulated recall. Ann Fam Med.

[7] Roland M, Everington S (2016). Tackling the crisis in general practice. BMJ.

[8] Hobbs FDR, Bankhead C, Mukhtar T, Stevens S, Perera-Salazar R, Holt T, Salisbury C (2016). Clinical workload in UK primary care: a retrospective analysis of 100 million consultations in England, 2007-14. Lancet.

[9] Lamb SE, Lall R, Hansen Z (2010). A multi-centred randomised controlled trial of a primary care-based cognitive behavioural programme for low Back pain. The Back skills training (BeST) trial. Health Technol Assess.

[10] Hollinghurst S, Sharp D, Ballard K (2008). Randomised controlled trial of Alexander technique lessons, exercise, and massage (ATEAM) for chronic and recurrent back pain: economic evaluation. BMJ.

[11] Hay EM, Mullis R, Lewis M (2005). Comparison of physical treatments versus a brief pain-management programme for back pain in primary care: a randomised clinical trial in physiotherapy practice. Lancet.

[12] UK BEAM Trial Team (2004). United Kingdom back pain exercise and manipulation (UK BEAM) randomised trial: effectiveness of physical treatments for back pain in primary care. BMJ.

[13] Oliveira Crystian B., Maher Chris G., Pinto Rafael Z., Traeger Adrian C., Lin Chung-Wei Christine, Chenot Jean-François, van Tulder Maurits, Koes Bart W. (2018). Clinical practice guidelines for the management of non-specific low back pain in primary care: an updated overview. European Spine Journal.

[14] Foster Nadine E, Anema Johannes R, Cherkin Dan, Chou Roger, Cohen Steven P, Gross Douglas P, Ferreira Paulo H, Fritz Julie M, Koes Bart W, Peul Wilco, Turner Judith A, Maher Chris G, Buchbinder Rachelle, Hartvigsen Jan, Cherkin Dan, Foster Nadine E, Maher Chris G, Underwood Martin, van Tulder Maurits, Anema Johannes R, Chou Roger, Cohen Stephen P, Menezes Costa Lucíola, Croft Peter, Ferreira Manuela, Ferreira Paulo H, Fritz Julie M, Genevay Stéphane, Gross Douglas P, Hancock Mark J, Hoy Damian, Karppinen Jaro, Koes Bart W, Kongsted Alice, Louw Quinette, Öberg Birgitta, Peul Wilco C, Pransky Glenn, Schoene Mark, Sieper Joachim, Smeets Rob J, Turner Judith A, Woolf Anthony (2018). Prevention and treatment of low back pain: evidence, challenges, and promising directions. The Lancet.

[15] National Institute for Health & Care Excellence (2014). NICE clinical guideline [CG177] osteoarthritis: care and management in adults.

[16] Foster NE, Hartvigsen J, Croft PR (2012). Taking responsibility for the early assessment and treatment of patients with musculoskeletal pain: a review and critical analysis. Arthritis Res Ther.

[17] Lin I, Wiles L, Waller R (2019). What does best practice care for musculoskeletal pain look like? Eleven consistent recommendations from high-quality clinical practice guidelines: systematic review Br J sports med published online first: 02.

[18] Imison C, Naylor C (2010). Referral management: lessons for success.

[19] Hagen KB, Smedslund G, Østerås N (2016). Quality of community based osteoarthritis care: a systematic review and meta-analysis. Arthritis Care Res.

[20] Downie A, Hancock M, Jenkins H (2019). How common is imaging for low back pain in primary and emergency care? Systematic review and meta-analysis of over 4 million imaging requests across 21 years Br J sports med published online first: 13.

[21] Ivanova JI, Birnbaum HG, Schiller M, Kantor E, Johnstone BM, Swindle RW (2011). Real-world practice patterns, health-care utilization, and costs in patients with low back pain: the long road to guideline-concordant care. Spine J.

[22] Williams CM, Maher CG, Hancock MJ, McAuley JH, McLachlan AJ, Britt H (2010). Low back pain and best practice care: a survey of general practice physicians. Arch Intern Med.

[23] Buchbinder R, van Tulder M, Oberg B, Costa LM, Woolf A, Schoene M (2018). Low back pain: a call for action. Lancet..

[24] Hill JC, Dunn KM, Lewis M, Mullis R, Main CJ, Foster NE (2008). A primary care back pain screening tool: identifying patient subgroups for initial treatment. Arthritis Rheum.

[25] Hill JC, Whitehurst DG, Lewis M, Bryan S, Dunn KM, Foster NE (2011). Comparison of stratified primary care management for low Back pain with current best practice (STarT Back): a randomised controlled trial. Lancet..

[26] Whitehurst DG, Bryan S, Lewis M, Hill J, Hay EM (2012). Exploring the cost-utility of stratified primary care management for low back pain compared with current best practice within risk-defined subgroups. Ann Rheum Dis.

[27] Main C, Hill JC, Sowden G, Watson P (2012). Integrating physical and psychosocial approaches to treatment in low back pain. The development and content of the Keele STarT Back trial's "high risk" intervention. Physiother.

[28] Sowden Gail, Hill Jonathan Charles, Morso Lars, Louw Quninette, Foster Nadine Elizabeth (2018). Advancing practice for back pain through stratified care (STarT Back). Brazilian Journal of Physical Therapy.

[29] Foster NE, Hill JC, Sowden G. Matching Patients to Treatments. Pain and Rehabilitation - J Physiother Pain Assoc. 01/2014; 2014(36).

[30] National Institute for Health and Clinical Excellence. Low back pain and sciatica in over 16s: assessment and management. NICE guideline. Guideline. 2016 30-Nov-16. 27929617

[31] Van Wambeke P, Desomer A, Ailliet L, Berquin A, Demoulin C, Dewachter J, et al. Summary: Low back pain and radicular pain: assessment and management. Belgian Health Care Knowledge Centre (KCE) Report 287Cs. Good Clin Pract (GCP) Brussels. 2017.

[32] The Bree Collaborative: Spine/Low Back Pain Topic. Report & Recommendations, November 2013. (accessed 16th Sept 2019) http://www.breecollaborative.org/wp-content/uploads/spine_lbp.pdf

[33] Mallen CD, Peat G, Thomas E, Dunn KM, Croft PR (2007). Prognostic factors for musculoskeletal pain in primary care: a systematic review. Br J Gen Pract.

[34] Henschke N, Ostelo RW, Terwee CB, van der Windt DA (2012). Identifying generic predictors of outcome in patients presenting to primary care with nonspinal musculoskeletal pain. Arthritis care & research.

[35] Hill JC, Afolabi EK, Lewis M, Dunn KM, Roddy E, van der Windt DA, Foster NE (2016). Does a modified STarT Back tool predict outcome with a broader group of musculoskeletal patients than back pain? A secondary analysis of cohort data. BMJ Open.

[36] Muller S, Thomas E, Dunn KM, Mallen CD (2013). A prognostic approach to defining chronic pain across a range of musculoskeletal pain sites. Clin J Pain.

[37] Artus M, Campbell P, Mallen CD, Dunn KM, van der Windt DA (2017). Generic prognostic factors for musculoskeletal pain in primary care: a systematic review. BMJ Open.

[38] Dunn KM, Campbell P, Lewis M, Hill JC, van der Windt DA, Afolabi E, Protheroe J, Wathall S, Jowett S, Oppong R, Mallen CD, Hay E, Foster NE. Refinement and validation of the Keele STarT MSK Tool for stratifying patients with musculoskeletal pain. [Submitted to PLOS Med]. 10.1002/ejp.1821 34101299

[39] Babatunde OO, Jordan JL, Van der Windt DA, Hill JC, Foster NE, Protheroe J (2017). Effective treatment options for musculoskeletal pain in primary care: a systematic overview of current evidence. PLoS One.

[40] Protheroe J, Saunders B, Bartlam B, Dunn KM, Cooper V, Campbell P, Hill JC, Tooth S, Mallen CD, Hay EM, Foster NE. Matching treatment options for risk sub-groups in musculoskeletal pain: a consensus groups study. BMC Musculoskelet Disord. 20(1):–271. 10.1186/s12891-019-2587-z PMC6545223 31153364

[41] Saunders B, Hill JC, Foster NE, Cooper V, Protheroe J, Chudyk A, Dunn KM, Chew-Graham C, Bartlam B. Feasibility of delivery of stratified primary care for patients with musculoskeletal pain: qualitative findings from the STarT MSK feasibility and pilot trial. [Submitted to BMC Fam Pract]. 10.1186/s12875-020-1098-1 PMC7014618 32046656

[42] Eldridge S, Kerry S, Torgerson DJ (2009). Bias in identifying and recruiting participants in cluster randomised trials: what can be done?. BMJ.

[43] Margham T (2011). Musculoskeletal disorders: time for joint action in primary care. Br J Gen Pract.

[44] Somerville Simon, Hay Elaine, Lewis Martyn, Barber Julie, van der Windt Danielle, Hill Jonathan, Sowden Gail (2008). Content and outcome of usual primary care for back pain: a systematic review. British Journal of General Practice.

[45] Maserejian Nancy N., Fischer Michael A., Trachtenberg Felicia L., Yu Jing, Marceau Lisa D., McKinlay John B., Katz Jeffrey N. (2013). Variations Among Primary Care Physicians in Exercise Advice, Imaging, and Analgesics for Musculoskeletal Pain: Results From a Factorial Experiment. Arthritis Care & Research.

[46] Craig P, Dieppe P, Macintyre S (2008). Developing and evaluating complex interventions: the new Medical Research Council guidance. BMJ.

[47] Kroenke Kurt, Krebs Erin E, Turk Dennis, Von Korff Michael, Bair Matthew J, Allen Kelli D, Sandbrink Friedhelm, Cheville Andrea L, DeBar Lynn, Lorenz Karl A, Kerns Robert D (2019). Core Outcome Measures for Chronic Musculoskeletal Pain Research: Recommendations from a Veterans Health Administration Work Group. Pain Medicine.

[48] Hill JC. Outcome Measures in Musculoskeletal Practice. Chapter 21 in Grieves’ Modern Musculoskeletal Physiotherapy, 4th edition. Jull et al. Elsevier. 2015.

[49] UK Government website. https://assets.publishing.service.gov.uk/government/uploads/system/uploads/attachment_data/file/464430/English_Index_of_Multiple_Deprivation_2015_-_Guidance.pdf. Accessed 16 Sept 2019.

[50] Roland M, Morris R (1983). A study of the natural history of back pain. Part I: development of a reliable and sensitive measure of disability in low-back pain Spine (Phila Pa 1976).

[51] BenDebba M, Heller J, Ducker TB, Eisinger JM (2002). Cervical spine outcomes questionnaire: its development and psychometric properties. Spine (Phila Pa 1976).

[52] MacDermid JC, Walton DM, Avery S, Blanchard A, Etruw E, McAlpine C, Goldsmith CH (2009). Measurement properties of the neck disability index: a systematic review. J Orthop Sports Phys Ther.

[53] Breckenridge JD, McAuley JH (2011). Shoulder pain and disability index (SPADI). J Physiother.

[54] Perruccio AV, Stefan Lohmander L, Canizares M, Tennant A, Hawker GA, Conaghan PG, Roos EM, Jordan JM, Maillefert JF, Dougados M, Davis AM (2008). The development of a short measure of physical function for knee OA KOOS-physical function Shortform (KOOS-PS) - an OARSI/OMERACT initiative. Osteoarthr Cartil.

[55] Ware JE (2000). SF-36 health survey update. Spine (Phila Pa 1976).

[56] Hill JC, Kang S, Benedetto E, Myers H, Blackburn S, Smith S, Dunn KM, Hay E, Rees J, Beard D, Glyn-Jones S, Barker K, Ellis B, Fitzpatrick R, Price A. Development and initial validation of the Arthritis Research UK Musculoskeletal Health Questionnaire (MSK-HQ) for use across musculoskeletal care pathways. BMJ Open. 2016. Aug 5;6(8):e012331. 10.1136/bmjopen-2016-012331 PMC4985936 27496243

[57] Archer KR, Phelps KD, Seebach CL, Song Y, Riley LH, Wegener ST (2012). Comparative study of short forms of the Tampa scale for Kinesiophobia: fear of movement in a surgical spine population. Arch Phys Med Rehabil.

[58] Holt N, Pincus T (2016). Developing and testing a measure of consultation-based reassurance for people with low back pain in primary care: a cross-sectional study. BMC Musculoskelet Disord.

[59] Herdman M, Gudex C, Lloyd A, Janssen M, Kind P, Parkin D, Bonsel G, Badia X (2011). Development and preliminary testing of the new five-level version of EQ-5D (EQ-5D-5L). Qual Life Res.

[60] Kamper SJ, Maher CG, Mackay G (2009). Global rating of change scales: a review of strengths and weaknesses and considerations for design. J Man Manip Ther.

[61] Morris NS, MacLean CD, Chew LD, Littenberg B (2006). The single item literacy screener: evaluation of a brief instrument to identify limited reading ability. BMC Fam Pract.

[62] Eldridge S (2016). How big should the pilot study for my cluster randomised trial be?. Stat Methods Med Res.

[63] Adams G, Gulliford MC, Ukoumunne OC, Eldridge S, Chinn S, Campbell MJ (2004). Patterns of intra-cluster correlation from primary care research to inform study design and analysis. J Clin Epidemiol.

[64] Eldridge SM, Ashby D, Kerry S (2006). Sample size for cluster randomized trials: effect of coefficient of variation of cluster size and analysis method. Int J Epidemiol.

[65] Vickers AJ (2003). How many repeated measures in repeated measures designs?. Stat Issues for comparative trials BMC Med Res Methodol.

