# Supplementary material for: Stratified primary care versus non-stratified care for musculoskeletal pain: findings from the STarT MSK feasibility and pilot cluster randomized controlled trial
Source: BMC Fam Pract. 2020 Feb 11;21:30. doi: 10.1186/s12875-019-1074-9 (PMC7014664; doi:10.1186/s12875-019-1074-9)
Supplement: Supplementary file 1 — Additional file 1. Summary of participant self-reported measures. [file 12875_2019_1074_MOESM1_ESM.docx]

**Additional file 1.** Summary of participant self-reported measures

| **Conceptual domain** | **Operational definition** | | | **Empirical measure used** | | **Number of items** | | **Time-point of data collection** | |
| --- | --- | --- | --- | --- | --- | --- | --- | --- | --- |
| **Patient descriptors** |  | | |  | |  | |  | |
| Age | Age at index consultation | | | Date of birth | | 1 | | GP EMR | |
| Sex | Sex | | | Male / Female | | 1 | | GP EMR | |
| Index pain location | Site of index pain complaint | | | Choice of anatomical region | | 1 | | GP EMR | |
| Pain intensity | Usual pain intensity | | | NRS 0-10 | | 1 | | GPEMR, I, 6FU, MF, MDC | |
| Socioeconomic status (IMD) | The individual’s (i) current or (ii) most recent job title | | | Job title - categorised as manual/non-manual | | 2 | | GP EMR | |
| GP Practice | GP Practice consulted for MSK pain | | | Taken from medical record | | 1 | | GP EMR | |
| Episode duration | Time since last whole month pain free | | | Episode duration | | 1 | | I | |
| Health Literacy Screen | Health literacy | | | Single question - Likert scale | | 1 | | I | |
| Comorbidities | Self-reported diagnosed comorbidities from a provided list | | | Yes | | 1 | | I | |
| Widespread pain | Presence of widespread pain | | | Yes / no | | 1 | | I | |
| Support needed | Support to complete questionnaire | | | Yes / no | | 1 | | I | |
| Living arrangements | Lives alone | | | Yes / no | | 1 | | I | |
| Previous episodes | Number of previous pain episodes | | | Number | | 1 | | I | |
| Perceived reassurance from GP consultation | Effective Consultation and Reassurance Questionnaire (ECRQ) | | | 12 items with 7-point Likert scale | | 12 | | I | |
| Receipt of written education material from GP | Single item to ask if patient received written information at their GP visit | | | Yes / no / don’t remember | | 1 | | I | |
| Pain self-efficacy | Single item - confidence to manage pain | | | NRS 0-10 | | 1 | | I, MF | |
| Psychological distress | Single item regarding level of distress | | | NRS 0-10 | | 1 | | I, MF | |
| Employment status and absence from work | Employment status at time of questionnaire | | | Yes/No and details | | 1 | | I, 6FU | |
| Risk status – development version of STarT MSK Tool | Risk of persistent disabling pain | | | Yes / No | | 9 | | I, 6FU | |
| Musculoskeletal health | Impact from MSK symptoms | | | MSK-HQ | | 14 | | I, 6FU | |
| Overall rating of change | Change since index pain consultation | | | Single question -5 to +5 scale | | 1 | | I, 6FU | |
| Physical activity level | Days past week of moderate activity | | | 1-7 days | | 1 | | I, 6FU | |
| Fear avoidance beliefs | Fear of movement | | | TSK-11 | | 11 | | I, 6FU | |
| Satisfaction | Satisfaction with care | | | Single question - Likert scale | | 1 | | I, 6FU | |
| Physical function  Back pain patients  Neck pain patients  Shoulder pain patients  Knee pain patients  Multi-site pain | |  |  | |  | |  | |  |
|  |  | Site specific physical function | | RMDQ – original version  NDI  SPADI  KOOS-PS  SF-12 PCS | | 24  10  13  7  12 | | I, 6FU  I, 6FU  I, 6FU  I, 6FU  I, 6FU | |
| Health-related quality of life | Utility-based quality of life | | | EuroQol-5D | | 5 | | I, 6FU MDC | |
| Healthcare costs  Performance at work  Work absence  Health care resource use |  | | |  | |  | |  | |
|  | How productivity at work is affected | | | 0-10 NRS | | 1 | | I, 6FU | |
|  | Number of days absent from work | | | Yes/No and details | | 1 | | I, 6FU | |
|  | Use of primary care, other NHS services, and private healthcare | | | Yes/No and if Yes details of resources used | | 3 | | 6FU | |
| GP EMR – GP EMR audit; I – initial participant questionnaire; 6FU – 6-month participant follow-up questionnaire; NRS – numerical rating scale. MF – monthly participant follow-up questionnaire. MDC – minimal data collection. | | | | | | | | | |
